# A Monocarbonyl Curcuminoid Derivative Inhibits the Activity of Human Glutathione Transferase A4-4 and Chemosensitizes Glioblastoma Cells to Temozolomide

**DOI:** 10.3390/ph17030365

**Published:** 2024-03-11

**Authors:** Steliana Tsouri, Evanthia Tselo, Georgios E. Premetis, Veronika Furlan, Panagiota D. Pantiora, Barbara Mavroidi, Dimitris Matiadis, Maria Pelecanou, Anastassios C. Papageorgiou, Urban Bren, Marina Sagnou, Nikolaos E. Labrou

**Affiliations:** 1 Laboratory of Enzyme Technology, Department of Biotechnology, School Applied Biology and Biotechnology, Agricultural University of Athens, 75 Iera Odos Street, 11855 Athens, Greece; stella.ts.18@gmail.com (S.T.); stud315088@aua.gr (E.T.); giorgos.prem@gmail.com (G.E.P.); or pantiora@aua.gr (P.D.P.); 2 Faculty of Chemistry and Chemical Engineering, University of Maribor, Smetanova 17, SI-2000 Maribor, Slovenia; veronika.furlan@um.si (V.F.); urban.bren@um.si (U.B.); 3 Institute of Biosciences & Applications, National Centre for Scientific Research “Demokritos”, 15310 Athens, Greece; bmavroidi@bio.demokritos.gr (B.M.); or dmatiadis@gmail.com (D.M.); pelmar@bio.demokritos.gr (M.P.); sagnou@bio.demokritos.gr (M.S.); 4 Department of Biomedical Sciences, University of West Attica, Egaleo Park Campus, 12243 Athens, Greece; 5 Turku Bioscience Centre, University of Turku and Åbo Akademi University, 20521 Turku, Finland; anapap@utu.fi; 6 Faculty of Mathematics, Natural Sciences and Information Technologies, University of Primorska, Glagoljaška 8, SI-6000 Koper, Slovenia; 7 Institute of Environmental Protection and Sensors, Beloruska Ulica 7, SI-2000 Maribor, Slovenia

**Keywords:** chemosensitization, chemoresistance, curcumin, glioblastoma, human glutathione transferase A4-4, ellagic acid, monocarbonyl curcumin derivatives, GST inhibition, temozolomide sensitization

## Abstract

Human glutathione transferase A4-4 (hGSTA4-4) displays high catalytic efficiency towards 4-hydroxyalkenals and other cytotoxic and mutagenic products of radical reactions and lipid peroxidation. Its role as a target for the chemosensitization of cancer cells has not been investigated so far. In this study, the inhibitory potency of twelve selected natural products and ten monocarbonyl curcumin derivatives against hGSTA4-4 was studied. Among natural products, ellagic acid turned out to be the strongest inhibitor with an IC_50_ value of 0.44 ± 0.01 μM. Kinetic analysis using glutathione (GSH) and 1-chloro-2,4-dinitrobenzene (CDNB) as variable substrates showed that ellagic acid behaved as a competitive inhibitor towards both GSH and CDNB, with K_i_ values of 0.39 ± 0.02 and 0.63 ± 0.03 μM, respectively. Among the curcumin derivatives studied, three proved to be the most potent inhibitors, in the order DM151 > DM101 > DM100, with IC_50_ values of 2.4 ± 0.1 μM, 12.7 ± 1.1 μΜ and 16.9 ± 0.4 μΜ, respectively. Further kinetic inhibition analysis of the most active derivative, DM151, demonstrated that this compound is a mixed inhibitor towards CDNB with inhibition constants of K_i_ = 4.1 ± 0.5 μM and K_i’_ = 0.536 ± 0.034 μM, while it is a competitive inhibitor towards GSH with a K_i_ = 0.98 ± 0.11 μM. Molecular docking studies were performed to interpret the differences in binding of ellagic acid and curcumin derivatives to hGSTA4-4. The *in silico* measured docking scores were consistent with the obtained experimental data. Hydrogen bonds appear to be the main contributors to the specific binding of monocarbonyl curcumin derivatives, while π-π stacking interactions play a key role in the enzyme–ellagic acid interaction. *In vitro* cytotoxicity assessment of the worst (DM148) and the best (DM151) inhibitors was performed against glioblastoma cell lines U-251 MG and U-87 MG. The results revealed that DM151 displays considerably higher cytotoxicity against both glioblastoma cell lines, while the glioblastoma cytotoxicity of DM148 was very limited. Furthermore, low and non-toxic doses of DM151 sensitized U-251 MG cells to the first-line glioblastoma chemotherapeutic temozolomide (TMZ), allowing us to propose for the first time that hGSTA4-4 inhibitors may be attractive therapeutic partners for TMZ to optimize its clinical effect in glioblastoma chemotherapy.

## 1. Introduction

Ultraviolet (UV) and ionizing radiation, heat, free radicals, hypoxia, xenobiotics, metal ions, and reactive oxygen or nitrogen species (ROS or RNS) are among the most widely studied external and internal inducers of cellular oxidative stress [1]. They heavily contribute to the excessive generation and accumulation of ROS, balancing out and overtaking all the natural antioxidant machinery of the cells [2]. One of the consequences of this imbalance is the free radical non-enzymatic peroxidation of polyunsaturated fatty acids (PUFAs) in cell membranes, leading to the formation of 4-hydroxy-2,3-trans-nonenal (4-hydroxynonenal, 4-HNE) [3]. 4-HNE is a potentially toxic molecule that may be involved in electrophilic, Michael, and Schiff type reactions with various macromolecules and can also act as a pro-apoptotic second messenger that alters cell cycle signaling pathways in a concentration-dependent manner [4,5]. It is important to note, however, that under physiological aerobic conditions (i.e., in the presence of basal levels of ROS), cells have been adapted to perform their function normally at basal levels of 4-HNE. In human blood and serum, this concentration has been estimated to be about 0.05–0.15 μM [6,7]. In contrast, 4-HNE levels can reach >100 µM when excessive lipid peroxidation occurs, which is usually detected in various pathological conditions, including myocardial and neurodegenerative diseases, metabolic syndrome, and cancer [3,8,9]. However, the accumulation of 4-HNE has also been related to the induction of defense mechanisms against oxidative stress and the protection of neighboring cells from apoptosis [10].

The maintenance of this sensitive and crucial intracellular level of 4-HNE is achieved by balancing oxidative 4-HNE production with its removal from the cells. This function is accomplished by the alpha-class glutathione transferases (GSTA). In particular, the hGSTA4-4 isoform catalyzes the conjugation of 4-HNE to GSH with high catalytic efficiency by exhibiting a much higher affinity for 4-HNE than for other xenobiotics, indicating the critical role of hGSTA4-4 in regulating 4-HNE homeostasis [11,12]. Notably, the expression level and degree of function of various GSTs have been associated with human cancers. Overexpression of GSTs has been reported in colon, kidney, pancreatic, and liver cancers, suggesting a cancer-promoting and drug-resistance-inducing effect [13,14,15,16,17]. Importantly, in the case of the GSTA family, a variety of cancer-related signaling pathways have been shown to be affected, suggesting that the occurrence and development of tumors are potentially subjected to their action. Accordingly, studies have shown that hGSTA4-4 can promote malignant progression of lung cancer, gastric cancer, colon cancer, and other tumors [18,19,20,21,22]. Furthermore, increased tumor invasion and migration observed in liver cancer have been related to high expression of hGSTA4-4, as a consequence of the induced Akt phosphorylation that promotes *in vivo* and *in vitro* tumor growth [23]. Regarding multidrug resistance (MDR), hGSTA1-1 has been shown to be strongly involved in the detoxification of chlorambucil by catalyzing the GSH conjugation reaction of this drug, thereby reducing its effectiveness. Similarly, resistance to bacitracin, melphalan, thiotepa, cyclophosphamide, imatinib, and cisplatin has been partially attributed to the activity of hGSTA1-1 [24].

Based on the aforementioned evidence, the search for specific inhibitors targeting GST isoenzymes is considered a potential therapeutic strategy for various diseases and cancer MDR reversal. More specifically, the anti-inflammatory agent sulfasalazine was found to effectively regulate GSH/GST-mediated drug resistance in melphalan-resistant cells and improve drug activity by inhibiting GST isoenzymes that belong to the alpha, mu, and pi classes [25]. Zou et al. revealed that hGSTA1-1 is a predominant GST isozyme associated with cisplatin resistance, and hGSTA1-1 silencing markedly increases cisplatin cytotoxicity in human gastric, ovarian, and lung cancer cell lines [26]. Similarly, some 2,2′-dihydroxybenzophenones were shown to have significant hGSTA1-1 inhibitory activity, which was also found to be related to their cytotoxicity against human colon adenocarcinoma [27,28]. Additionally, the natural flavonoids fisetin and myricetin have been reported to act as effective hGSTA1-1 inhibitors [29,30]. Finally, a series of 34 monocarbonyl curcumin analogues, including 2,6-dibenzylidenecyclohexanones, 2,5-dibenzylidenecyclopentanones, and 1,4-pentadiene-3-ones, were evaluated as potential human GST inhibitors against hGSTA1-1, hGSTM1-1, and hGSTP1-1 isoenzymes [31]. hGSTA1-1 and hGSTM1-1 were inhibited more effectively by the curcumin analogs tested, whereas seven compounds inhibited hGSTA1-1 more strongly than curcumin [32].

Curcumin is the primary constituent of turmeric (*Curcuma longa*) extract, which contains numerous active compounds, among which curzerene, a sesquiterpene, has been shown to downregulate the expression of human hGSTA1-1 in lung cancer, resulting in tumor inhibition [33]. Curcumin is also among the very few reported compounds that inhibit *in vitro* the expression of hGSTA4-4 [34]. Interestingly, treatment of glioblastoma cells with curzerene resulted in lower expression of hGSTA4 mRNA and protein leading to a time-and dose-dependent manner inhibition of the proliferation, invasion, and migration of the cells. *In vivo*, after curzerene administration in glioblastoma-bearing nude mice, significant tumor growth inhibition and life extension were observed.

Here, we report an inhibition study of 12 natural products and ten monocarbonyl curcumin derivatives against hGSTA4-4. To the best of our knowledge, this is the first specific hGSTA4-4 enzyme inhibition study reported. This can be witnessed by the lack of publications in both the PubMed^®^ and the Reaxys^®^ databases (searched on 1 March 2024) under the search for “hGSTA4-4 inhibitors,” “hGSTA4-4 inhibition,” “glutathione-S-transferase A4-4 inhibitors”, and “glutathione-S-transferase A4-4 inhibition” in the title, abstract and keywords sections. S-(2-Iodobenzyl)-GSH was used by Bruns et al. as a potential inhibitor in crystallographic studies of hGSTA4-4 [35] without any specific enzymatic analysis, whereas Groom et al. evaluated dinitronaphthalene analogues against various GSTs and no hGSTA4-4 inhibition was observed [36]. In our study, the inhibition potential of the natural products (NPs) resveratrol, colchicine, gallic acid, safranal, coumaric acid, naringenin, polydatin, taxifolin hydrate, curcumin, quercetin, epigallocatechin gallate, and ellagic acid (Table 1), at a concentration of 100 μM, was evaluated. Similarly, a small library of monocarbonyl curcumin analogs [37] was also evaluated against hGSTA4-4 at the same concentration (Table 2). The most active compounds were studied in more detail to assess their inhibitory potency (IC_50_ and K_i_ values), and type of inhibition. Computational studies were also performed to identify the structural and molecular determinants of the enzyme–inhibitor interaction. Moreover, U-251 MG and U-87 MG glioblastoma cells were treated with the most and least active monocarbonyl curcumin derivatives to evaluate their cytotoxicity and potentially relate this function to their hGSTA4-4 inhibition potency. Finally, the potential of the two compounds to act synergistically with the first-line glioblastoma chemotherapeutic temozolomide was evaluated in U-251 MG glioblastoma cells.

## 2. Results and Discussion

### 2.1. Cloning, Expression, and Purification of hGSTA4-4 from Recombinant E. coli Cells

**Figure 1:**
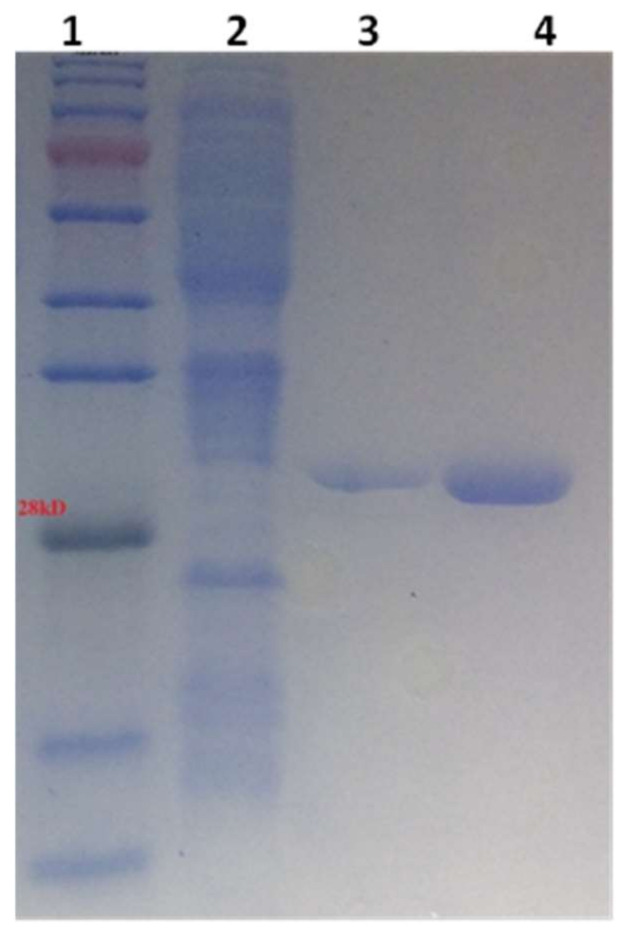
SDS-PAGE analysis of purified hGSTA4-4. Purification was achieved using metal ion affinity chromatography on Ni^2+^-IDA-Sepharose column. Lane 1: protein markers; Lane 2: crude extract of *E. coli* BL-21(DE3) transformed with pETite-hGSTA4-6His vector after induction with IPTG (1 mM). Lane 3: Purified hGSTA4-4. Elution was performed using 50 mM NaH_2_PO_4_ buffer containing 300 mM NaCl and 150 mM imidazole. Lane 4: Purified hGSTA4-4. Elution was achieved using 50 mM NaH_2_PO_4_ buffer containing 300 mM NaCl and 250 mM imidazole.

Recombinant hGSTA4-4 was expressed in *E. coli* BL21 (DE3) cells and purified to apparent homogeneity (Figure 1) with a 58% yield using affinity chromatography on Ni^2+^-IDA-Sepharose.

### 2.2. Screening of Natural Polyphenols as hGSTA4-4 Inhibitors

The inhibitory activity of 12 natural products, mostly polyphenols, was assessed at an initial concentration of 100 μM with the aim of identifying molecules that cause high hGSTA4-4 (%) inhibition. The potencies of the tested compounds are listed in Table 1. Resveratrol was the least potent inhibitor, exhibiting only 8.9% inhibition, whereas ellagic acid inhibited the enzyme almost completely, reaching a 95% inhibition. The second weakest inhibitor, colchicine, showed 17% inhibition, followed by some medium-potency inhibitors (gallic acid, safranal, coumaric acid, naringenin, polydatin, and taxifolin hydrate). The latter shows the highest % inhibition in the natural product group of medium inhibitory activity, reaching a value of 40%. Curcumin, quercetin, epigallocatechin gallate, and ellagic acid were the four most potent inhibitors, with inhibition ranging between 81 and 95%.

**Table 1:** Screening of the inhibitory potency of selected natural products against hGSTA4-4 at an initial concentration of 100 μM. No enzyme inhibition was observed in the absence of the compounds. The results are the mean of three individual experiments. Standard deviation was <5% in all cases.

Natural Products	Enzyme Inhibition (%)	Natural Products	Enzyme Inhibition (%)
Polydatin 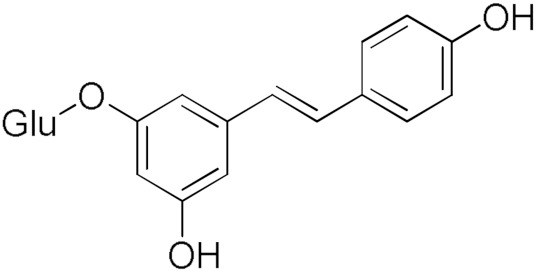	32.30	Resveratrol 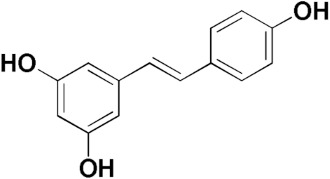	8.87
Taxifolin hydrate 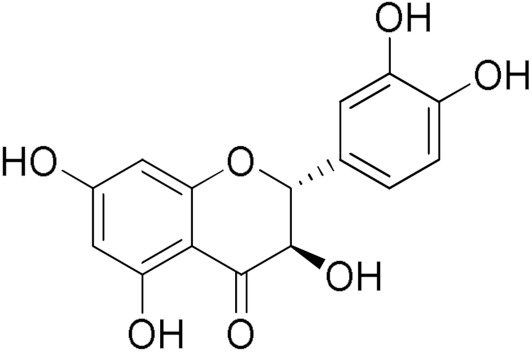	40.37	Curcumin 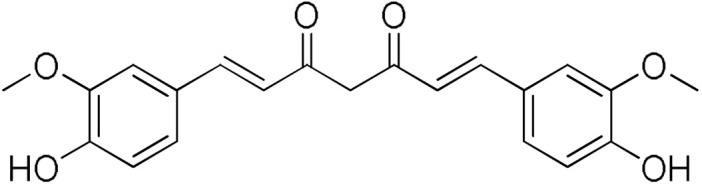	80.92
Naringenin 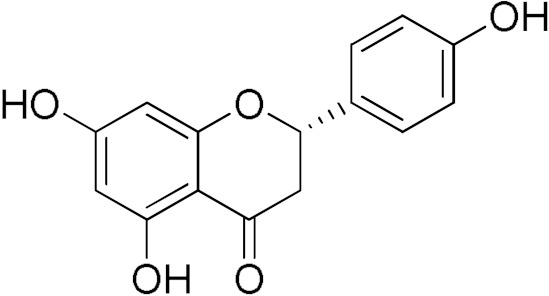	28.49	Safranal 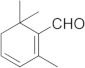	21.84
Epigallocatechin gallate 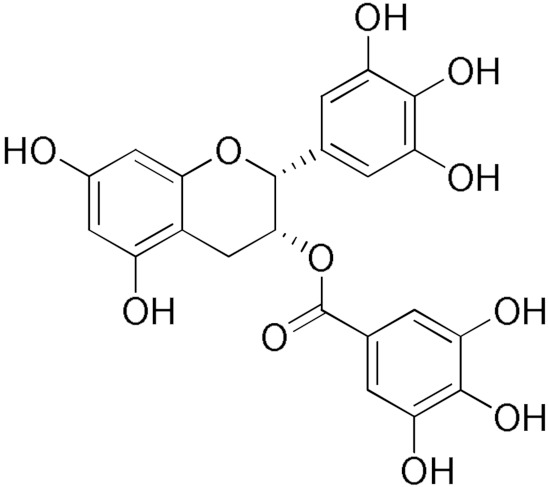	86.39	Gallic acid 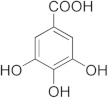	20.81
Quercetin 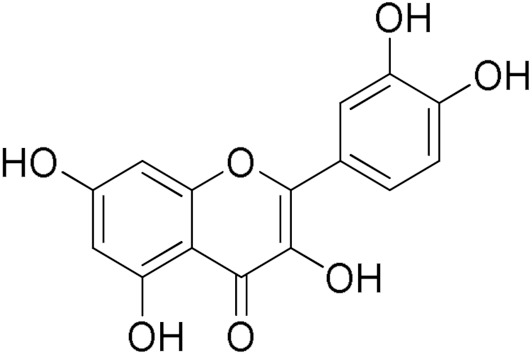	84.65	Coumaric Acid 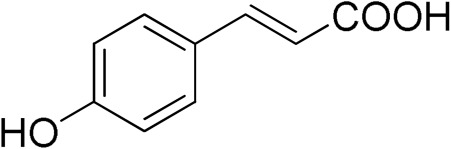	27.3
Colchicine 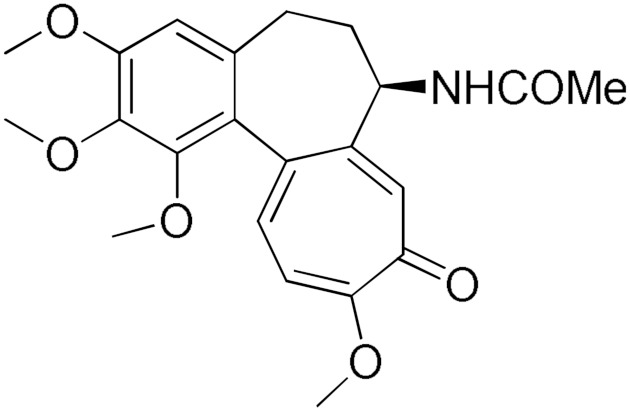	17.1	Ellagic Acid 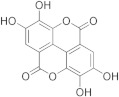	95.1

From a structural point of view, it is very interesting to note that resveratrol is the only one of the tested polyphenols which does not contain any carbonyl functional group. The addition of the glucose moiety to one of the resveratrol phenols to obtain the structure of polydatin resulted in a four-fold increase in the inhibition (%). In this case, the hemiacetal nature of the ring may potentiate the blocking of enzyme function. This may come to no surprise based on the fact that the major 4-HNE metabolite has been reported to be hemiacetalized 4-HNE/GSH, which may allow for the polydatin glucose hemiacetal part to interact with the enzyme [38,39]. Despite the presence of an unsaturated ketone in the compact colchicine structure, the low inhibition potential may be related to the lack of hydroxyl groups. On the other hand, in the case of the polyhydroxylated and structurally related taxifolin hydrate, quercetin, epigallocatechin gallate, and ellagic acid, the enzyme inhibition increases dramatically.

**Figure 2:**
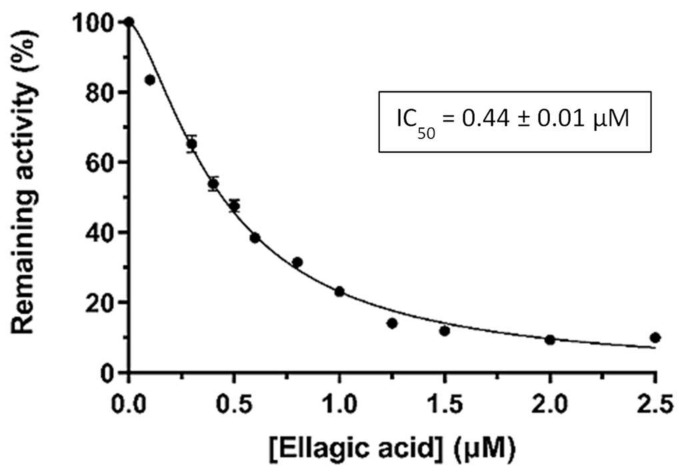
Concentration–response curve for the determination of IC_50_ value of ellagic acid against hGSTA4-4.

Ellagic acid, the best inhibitor in this initial screening, was selected for further dose–response studies and IC_50_ determination. Figure 2 shows the effect of varying ellagic acid concentrations on the enzyme activity. The concentration of ellagic acid which caused a 50% GSTA4-4 inhibition was determined to be IC_50_ = 0.44 ± 0.01 μΜ. This sub-micromolar value indicated a highly potent inhibitor.

### 2.3. Screening of Curcumin Derivatives as hGSTA4-4 Inhibitors

The search for effective hGSTA4-4 inhibitors and the rather high inhibitory potential exhibited by curcumin (Table 1) prompted us to explore the activity of some synthetic monocarbonyl curcumin derivatives. In general, monocarbonyl analogs of curcumin (MACs) have been designed and used to improve the chemical and metabolic stability, bioavailability, intestinal permeability, and water solubility of curcumin [40,41,42] while retaining or even increasing their efficacy in various biological systems compared to the mother compound. The results summarized in Table 2 allowed for the identification of three distinct groups of inhibitory activities. The lowest inhibition potency was observed for DM148, DM57, and DM95, with inhibition ranging from 4.6% to 55.7%. Compounds DM62, DM109, and DM46 exhibited medium inhibition potency (64.5–81.8%), whereas compounds MD96, DM151, DM100, and DM101 showed superior activity than curcumin and the highest potency among all tested derivatives (>90% inhibition).

**Table 2:** Screening of the inhibitory potency of curcumin derivatives against hGSTA4-4 at an initial concentration of 100 μM. No enzyme inhibition was observed in the absence of the compounds. The results are the mean of three individual experiments. Standard deviation was <5% in all cases.

Compound	Enzyme Inhibition (%)	Compound	Enzyme Inhibition (%)
**DM148** 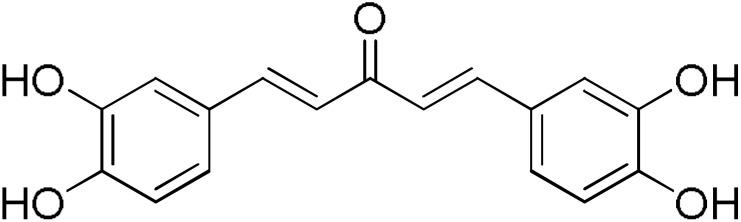	4.6	**DM62** 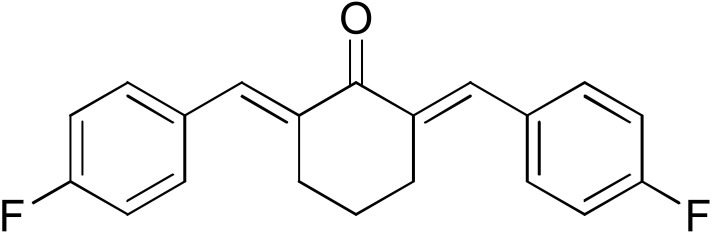	74.9
**DM95** 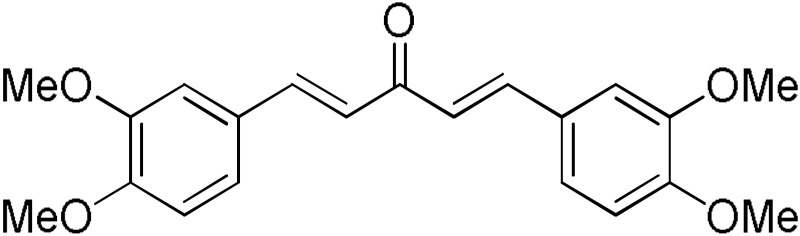	54.8	**DM96** 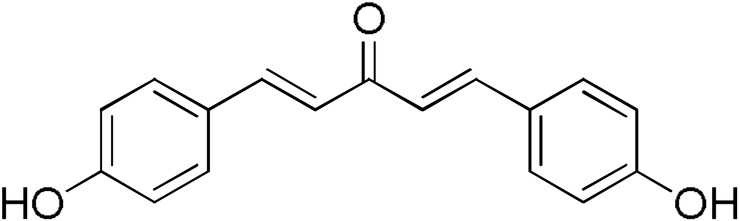	81.8
**DM57** 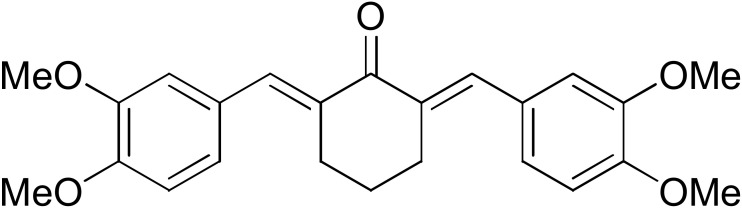	55.7	**DM151** 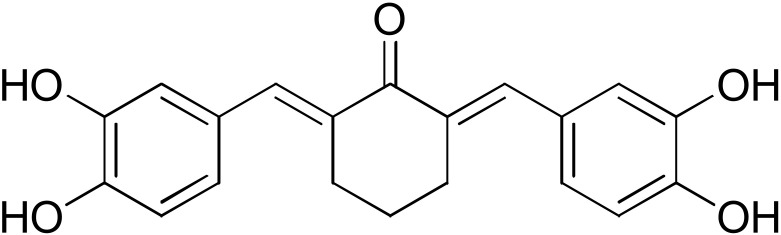	91.6
**DM46** 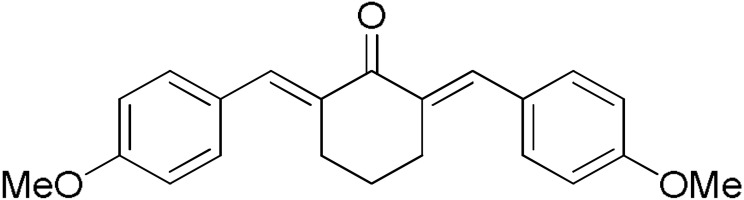	64.9	**DM101** 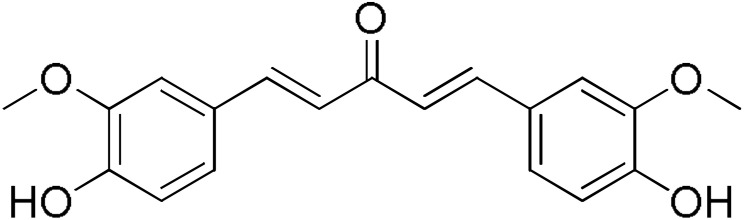	94.1
**DM109** 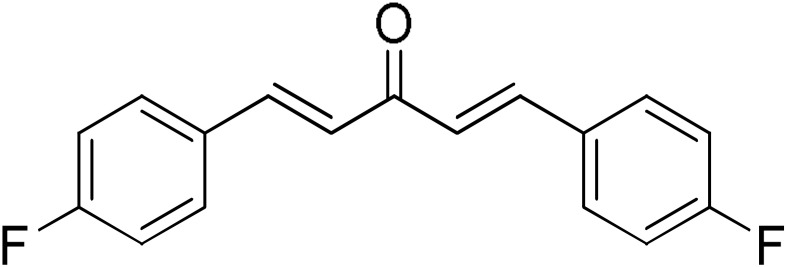	73.9	**DM100** 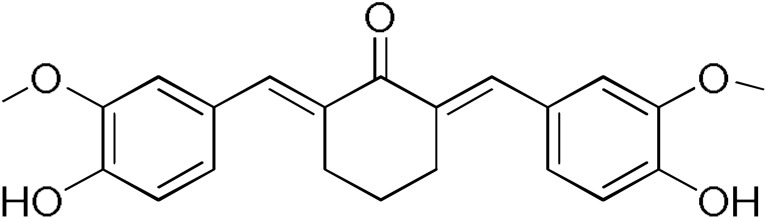	95.1

In our previous study, the activity of these molecules against the hGSTP1-1 isoenzyme was determined, and structural features that favor inhibitory potency were identified [37]. In the case of the hGSTA4-4 inhibition study, there are also important structure–activity relationships to be deduced. Curcumin was found to be a more effective inhibitor of the hGSTA4-4 isoform than of hGSTP1-1. In agreement with our previous findings, the presence of phenolic hydroxyls increases the potency, whereas the fluoro-substituted derivatives were better inhibitors than the methoxy derivatives and worse inhibitors than their phenolic counterparts. For all substitution patterns, the % inhibition value of cyclohexanone was consistently slightly higher than that of the corresponding acetone derivative. This is in direct contrast to the results obtained for the hGSTP1-1 enzyme, implying that the increased hydrophobicity, rigidity, and space occupancy of the cyclohexanone-bearing compounds favored the blockage of enzymatic activity.

The results of this initial screening of the performance of DM148 and DM151 under these experimental conditions are of great interest. Both compounds contain a 3,4-dihydroxy substitution pattern, and the effect of the central core of the molecule is detrimental. More specifically, the acetone-derived DM148 compound was the least active, whereas the cyclohexanone-containing DM151 monocarbonyl derivative was among the most active inhibitor with >90% inhibition. The inhibitory potential against hGSTP1-1 was not significantly different. Similarly, when assessed as potential tyrosinase, thioredoxin reductase, aldose reductase, and HIV integrase inhibitors, their IC_50_ values are very close [43,44,45,46]. This extreme difference in activity of the two compounds against hGSTA4-4 suggests the involvement of the middle linker unit in the diphenolic substitution pattern.

**Figure 3:**
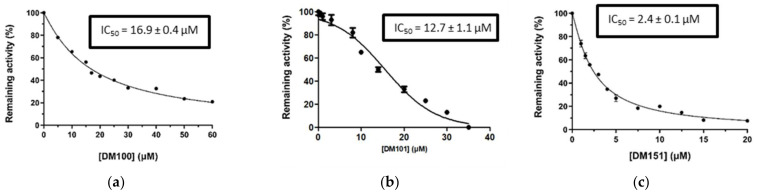
Concentration–response curves for the determination of the IC_50_ values of DM100 (**a**), DM 101 (**b**), and DM151 (**c**) against hGSTA4-4. The results represent the mean of three individual experiments.

Since DM151, DM100, and DM101 achieved very similar inhibition at this fairly high concentration, a dose–response study and IC50 determination for all three compounds were performed to identify the most potent of the three compounds. From the graphical representation of these results shown in Figure 3, the IC_50_ values derived for DM100, DM101, and DM151 are equal to 16.9 ± 0.4 μΜ, 12.7 ± 1.1 μΜ, and 2.4 ± 0.1 μΜ, respectively. Therefore, DM151 was found to be the most efficient inhibitor and selected for further investigation.

### 2.4. Kinetic Inhibition Studies of hGSTA4-4 with Ellagic Acid and DM151

The most potent inhibitors of hGSTA4-4, ellagic acid, and DM151, were further evaluated using kinetic inhibition studies. This would enable the determination of the type of inhibition caused by the tested compounds and their mode of binding to the target enzyme.

#### 2.4.1. Kinetic Inhibition Studies of hGSTA4-4 with Ellagic Acid

**Figure 4:**
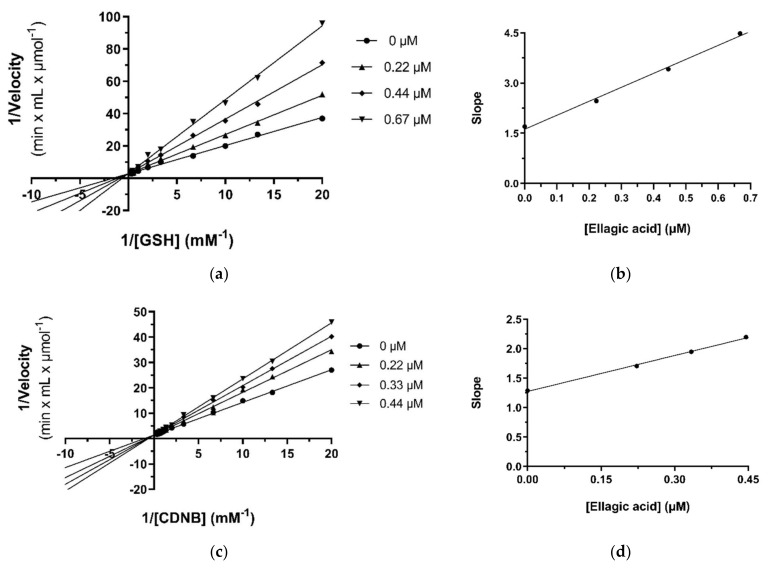
Kinetics of hGSTA4-4 inhibition by ellagic acid. (**a**) Lineweaver–Burk of the enzyme inhibition at different constant concentrations of ellagic acid (0, 0.22, 0.44, and 0.67 μM), when GSH was used as a variable substrate (0–2.5 mM). (**b**) Secondary plot of the slopes of each Lineweaver–Burk line as a function of ellagic acid concentration. (**c**) Lineweaver–Burk of the enzyme inhibition at different constant concentrations of ellagic acid (0, 0.22, 0.33, and 0.44 μM), when CDNB was used as a variable substrate (0–3 mM). (**d**) Secondary plot of the slopes of each Lineweaver–Burk line as a function of ellagic acid concentration.

In the presence of variable GSH concentrations, ellagic acid acts as a competitive inhibitor. This is indicated by the intersecting pattern of the double reciprocals (Lineweaver–Burk) shown in Figure 4a and the derived secondary plot (Figure 4b). These findings suggest that ellagic acid competes with GSH, with an inhibition constant of K_i_ = 0.39 ± 0.02 μM. The same competitive inhibitory behavior was observed when CDNB was used as the variable substrate (Figure 4c,d), with a calculated inhibition constant K_i_ = 0.63 ± 0.03 μΜ. The small difference between the inhibition constants (K_i_) for GSH and CDNB indicates that ellagic acid tends to bind with slightly higher affinity at the G-site than at the H site.

#### 2.4.2. Kinetic Inhibition Studies of hGSTA4-4 with DM151

**Figure 5:**
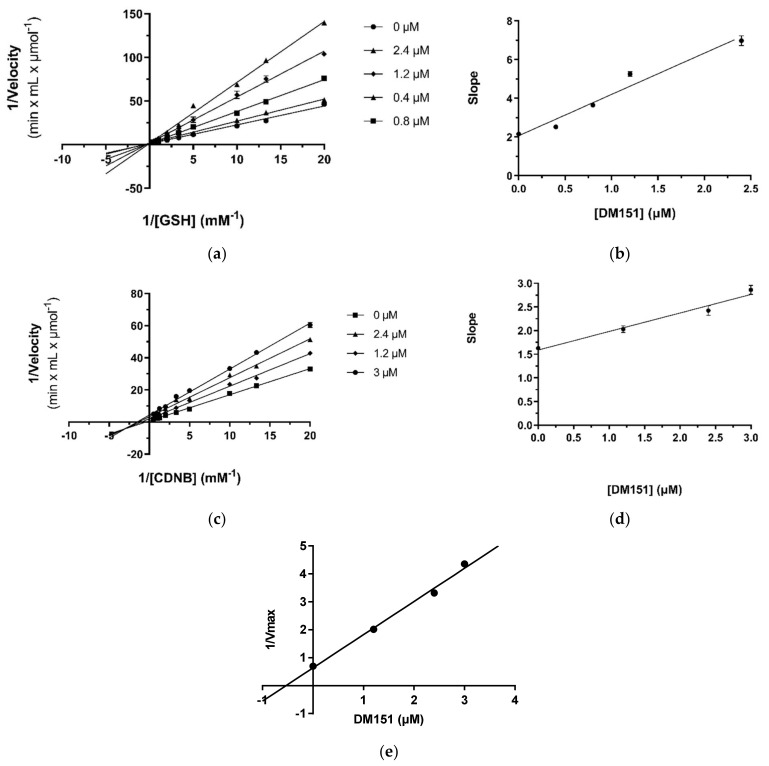
Kinetic inhibition of hGSTA4-4 by DM151. (**a**) Lineweaver–Burk of the enzyme inhibition at different constant concentrations of DM151 (0, 0.4, 0.8, 1.2, and 2.4 μM), when GSH was used as a variable substrate (0–4 mM). (**b**) Secondary plot of the slopes of each Lineweaver–Burk line as a function of DM151 concentration. (**c**) Lineweaver–Burk of the enzyme inhibition at different constant concentrations of DM151 (0, 1.2, 2.4, and 3 μM), when CDNB was used as a variable substrate (0–2 mM). (**d**) Secondary plot of the slopes of each Lineweaver–Burk line as a function of DM151 concentrations (**e**) Secondary plot of the 1/V_max_ obtained from Lineweaver–Burk lines as a function of DM151 concentrations.

Kinetic inhibition studies were also performed with DM151 to determine both the binding mode to hGSTA4-4 and the type of observed inhibition. In the presence of variable concentrations of GSH, DM151 behaves as a competitive inhibitor for GSH (Figure 5a), indicating that DM151 and GSH compete for the same binding site on the enzyme. To calculate the inhibition constant K_i_, a secondary graph of the slopes was plotted as a function of the inhibitor concentration (Figure 5b). The inhibition constant K_i_ = 0.98 ± 0.11 µM was estimated. When CDNB was used as a variable substrate, the Lineweaver–Burk lines intersected in quadrant III (Figure 5c). This behaviour shows that DM151 acts as a mixed-type inhibitor of CDNB, suggesting that DM151 binds to the enzyme at a different position from that of CDNB and forms a non-catalytic complex without prohibiting the binding of the substrate to the complex. Therefore, DM151 can bind to both the free enzyme and enzyme–CDNB complex. The inhibition constant for the binding of DM151 to the free hGSTA4-4 was calculated to be K_i_ = 4.1 ± 0.5 µM (Figure 5d), and to the hGSTA4-4–CDNB complex K_i_^’^ = 0.53 ± 0.03 μM (Figure 5e).

Interestingly, similar kinetic inhibition studies of the hGSTP1-1 isoform identified DM151 as a purely non-competitive inhibitor of both GSH and CDNB substrates [37]. In addition, when GSH was used as the variable substrate in the hGSTP1-1 study, the calculated inhibition constant was K_i_ = 5.79 µM which is much higher than the K_i_ = 0.98 µM inhibition constant calculated for the hGSTA4-4 isoform. In the case of the CDNB substrate, the K_i_ difference was only two fold, namely 9.55 and 4.1 µM for hGSTP1-1 and hGSTA4-4, respectively. Overall, these comparative results suggest a degree of selectivity for the GST inhibitory activity of DM151, making it a better inhibitor of hGSTA4-4 overexpressing systems and potentially a more effective therapeutic agent against hGSTA4-4 related pathologies.

A collective summary of the kinetic analysis results for ellagic acid and monocarbonyl curcuminoids is presented in Table 3.

### 2.5. Molecular Docking of Ellagic Acid, DM151, DM101, and DM100 to hGSTA4-4

The binding modes of ellagic acid, DM151, DM101, and DM100 at the active site of hGSTA4-4 were investigated using molecular docking with the CANDOCK algorithm and scoring function RMR6 [47]. The binding poses of ellagic acid, DM151, DM101, and DM100 at the active site of hGSTA4-4 were selected based on the lowest docking score values (Table 4). All docking scores were below −30 arbitrary units, indicating decent binding affinity of the studied inhibitors to hGSTA4-4. Ellagic acid and DM151 exhibited a higher affinity (lower docking score values) for hGSTA4-4 than DM101 and DM100 (higher docking score values). Furthermore, the order of the docking score values was in agreement with the experimental results, confirming the inhibitory activity of the studied compounds on hGSTA4-4.

**Figure 6:**
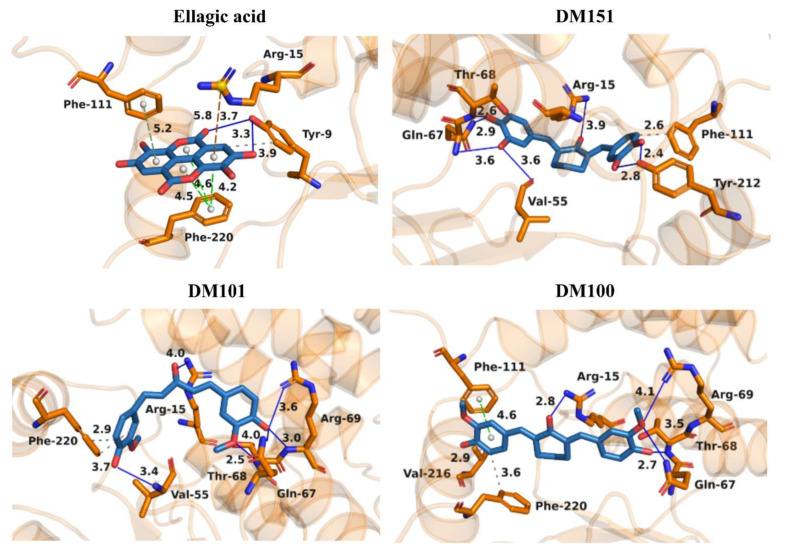
Binding modes of ellagic acid, DM151, DM101, and DM100 at the active site of hGSTA4-4. Carbon atoms of the studied inhibitors are depicted in light blue, while the carbon atoms of hGSTA4-4 amino acid residues are presented in orange. Oxygen atoms are red, and nitrogen atoms are dark blue. Hydrogen bonds are depicted with dark blue lines, hydrophobic interactions with gray dashed lines, π-cation interactions with orange dashed lines, and *π-π* stacking interactions with green dashed lines. To improve clarity, hydrogen atoms were omitted.

The Protein Ligand Interaction Profiler (PLIP) was used to evaluate the most important interactions between the four studied inhibitors and hGSTA4-4 [48]. In Figure 6, the most important interactions of ellagic acid, DM151, DM101, and DM100 with the binding site amino acid residues of hGSTA4-4 are presented. The binding poses indicate that all studied compounds form non-bonded interactions with Arg15. More specifically, ellagic acid forms a π-cation interaction at a distance of 5.82 Å, and curcumin derivatives DM151, DM101, and DM100 form hydrogen bonds at distances 3.88 Å, 4.04 Å, and 2.78 Å, respectively. The strongest hGSTA4-4 inhibitor, ellagic acid, is additionally stabilized through π-π stacking interactions with residues Phe220 and Phe111 at distances 4.22 Å, 4.47 Å, 4.58 Å, and 5.19 Å, respectively.

Ellagic acid also forms two hydrogen bonds with Tyr9 at distances 3.31 Å and 3.73 Å, respectively. The second strongest inhibitor DM151 is additionally stabilized through six hydrogen bonds with nearby residues Val55, Gln67, Thr68, and Tyr212 at distances of 3.62 Å, 3.60 Å, 2.91 Å, 2.60 Å, 2.77 Å, and 2.40 Å, respectively, as well as through a hydrophobic interaction with Phe111 at a distance of 2.60 Å. Moreover, DM101 and DM100 are both stabilized by hydrogen bonds with Gln67 (4.05 Å and 3.46 Å, respectively), Thr68 (2.53 Å and 2.65 Å, respectively), and Arg69 (3.58 Å and 4.07 Å, respectively) as well as hydrophobic interactions with Phe220 (2.93 Å, 3.72 Å, and 3.60 Å, respectively), while additional hydrogen bond with Val55 (3.44 Å) can be observed in the case of DM101 and additional π-π stacking interaction with Phe111 (4.59 Å) in the case of DM100. Hydrogen bonds, therefore, appear to play a crucial role in the inhibitory activity of the studied monocarbonyl curcumin derivatives, while *π-π* stacking interactions contribute the most to stabilizing ellagic acid in the binding pocket of hGSTA4-4. This result may be closely related to the apparent strongest inhibitory potency of ellagic acid among the studied compounds. Moreover, molecular docking results revealed that DM151 represents a stronger inhibitor of hGSTA4-4 (docking score: −44.59 arb. units) than of hGSTP1-1 enzyme (docking score: −36.99 arb. units) [37], indicating its potential as an effective therapeutic agent against hGSTA4-4-related pathologies. The difference in the binding affinity could be attributed to the higher number of hydrogen bonds (6) between DM151 and the amino acid residues Val55, Gln67, Thr68, and Tyr212 of hGSTA4-4, which were not observed in DM151 binding to the active site amino acid residues of hGSTP1-1 [37]. It is also important to note that nearby active site amino acid residues contribute to the binding of the studied inhibitors, which indicates that all the investigated compounds occupy the G-site at the N-terminal region of hGSTA4-4. This is in agreement with our previous study on monocarbonyl curcumin analogs as hGSTP1-1 inhibitors [37].

### 2.6. Cytotoxicity Studies of DM148 and DM151 against Glioblastoma Cells

Previous work on the effect of ellagic acid against U-87 MG glioblastoma cells reported the IC_50_ value of the compound to be equal to 91.2 µM after a 48 h treatment, suggesting that the natural polyphenol exhibits low glioblastoma cell toxicity [49]. Taking into consideration the submicromolar IC_50_ value obtained from the kinetic and inhibition studies against hGSTA4-4, it may be suggested that decreased cellular stability and very limited water solubility are the predominant factors for the decrease in cell toxicity [50].

**Figure 7:**
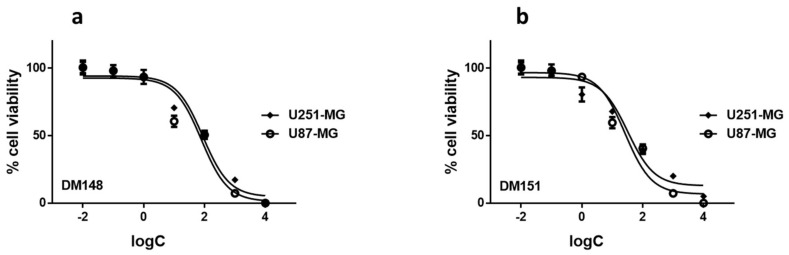
Dose–response curves against U251-MG and U87-MG cells cultured for 48 h with various concentrations (10^−3^ and 10^−8^ M) of compounds DM148 (**a**) and DM151 (**b**). All data are representative of at least three independent experiments.

Following our initial observation that DM148 barely inhibited the enzyme, whereas the DM151 was the most potent inhibitor of all tested monocarbonyl compounds, exhibiting a low micromolar IC_50_ value of 2.4 μΜ, their cytotoxicity effect against two glioblastoma cell lines, namely U-251 MG and U-87 MG, was evaluated. Both cell lines have been previously reported to overexpress hGSTA4-4, and downregulation by curzerene resulted in growth and invasion inhibition [34]. Similarly, it was hypothesized that this extreme difference in hGSTA4-4 inhibitory potential between DM148 and DM151 could also differentiate their cytotoxic effects. The IC_50_ values of the two compounds in the two glioblastoma cell lines are summarized in Table 5, and the sigmoidal decrease in % cell viability in the two cell lines after 48 h treatment with increasing concentrations of either of the monocarbonyl derivatives is depicted in Figure 7.

For DM148, the IC_50_ values (concentration of the drug resulting in a 50% reduction in cell viability) in U-251 MG and U-87 MG cell lines were 89.2 and 91.1 µM, respectively. Following a similar trend, for the cell toxicities of DM151, the same cell lines were 25.5 and 18.9 µM, respectively. It is very interesting to note that in both cell lines, the weaker hGSTA4-4 inhibitor DM148 was significantly less cytotoxic than its counterpart DM151. Assuming that the difference in lipophilicity does not affect the membrane permeability of the two compounds to a great extent, these results suggest that effective hGSTA4-4 inhibition and the disturbance of 4-HNE balance may be worth further investigation in the fight against glioblastoma. This is further supported by the fact that the median IC_50_ value for TMZ in U-87 MG cells at 48 h has been reported to be equal to 223.1 μM (IQR 92.0–590.1 μM) [51], which shows that this clinically used drug is nearly ten times less active *in vitro* than our monocarbonyl hGSTA4-4 inhibitors.

**Figure 8:**
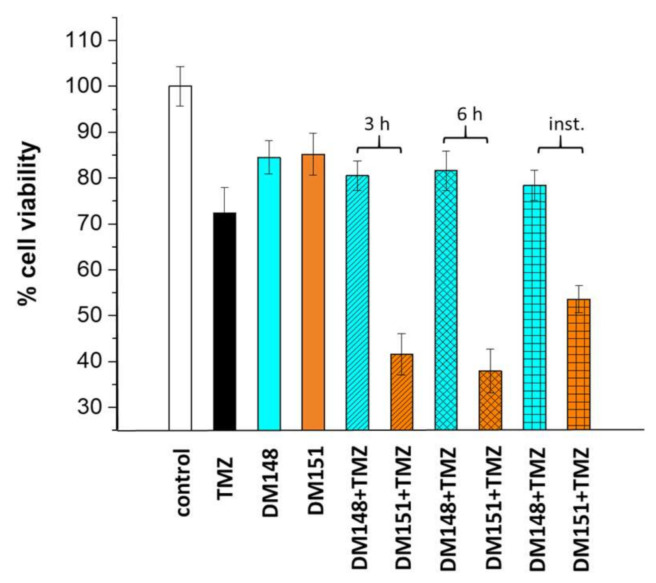
Effect on cell viability of DM148 (10 μM), DM151 (10 μM), and TMZ (150 μM) on U-251 MG cells. The cells were treated for 48 h with each of the compounds alone or pretreated with DM148 or DM151 for 0 h (inst.), 3, or 6 h, followed by TMZ treatment for 48 h.

TMZ has been the first-line chemotherapeutic agent for glioblastoma for more than two decades. However, its clinical use is limited by the induction of drug resistance and elimination of response. O^6^- methylguanine-DNA methyltransferase (MGMT) is the major TMZ resistance pathway, and its cellular levels have been correlated with patient survival [52]. Moreover, nuclear factor erythroid 2-related factor 2 (NRF2) and glutathione transferases have also been studied as mediators of TMZ resistance in glioblastoma cells [53]. Consequently, modulation of resistance-inducing cellular pathways has been shown to increase TMZ therapeutic potency [54]. This prompted us to explore whether DM148 or DM151 affects TMZ cytotoxicity in glioblastoma cells. Figure 8 shows how the % cell survival of U-251 MG cells is altered by treating the cells with a non-toxic concentration of DM148, DM151 (10 μΜ), or TMZ (150 μM) alone or in combination. When cells were treated for 48 h with any of the three agents individually, cell survival remained fairly high (>85%), indicating that the chosen concentration was not toxic. The treatment of cells with both TMZ and DM148 did not result in any dramatic changes in the percentage of cell survival. This result was independent of whether cells were pre-incubated for 3 or 6 h with DM148 or both compounds were added to the cell culture at the same time and incubated for 48 h. In contrast, a significant decrease in cell survival was observed when DM151 was combined with TMZ. The DM151 effect on TMZ cell cytotoxicity was also found to be time-dependent. Specifically, cell survival decreased from 85% to 55% when both compounds were added together. However, a more severe reduction in the cell population was observed when the cells were pretreated with DM151 for 3 h, when cell survival reached 45%. Longer pre-incubation of the cells with DM151 sensitized the cells even more to the action of TMZ, leading to a further reduction in living cells and a cell survival of approximately 35%.

Our results suggest that hGSTA4-4 may be implicated in TMZ sensitization and are in good agreement with the literature. Rocha et al. [55] demonstrated that TMZ treatment resulted in the induction of NRF2 and GSTs and was accompanied by an increase in GSH concentration in glioblastoma cells. This was further supported by the increased TMZ-induced DNA damage and cell death observed upon treatment with the GSH synthesis inhibitor L-buthionine [S,R]-sulfoximine (BSO) [55]. In another study on T98G, a GBM cell line with pre-existing TMZ resistance, increased glycolysis and hGSTM3-3 expression were observed at basal levels, whereas hGSTM3-3 knockdown resulted in increased TMZ toxicity and decreased invasion ability [56]. Based on our findings that pre-incubation of glioblastoma cells for 3 and 6 h with DM151 makes cells more susceptible to TMZ by increasing its cell killing effect, it would be of great interest to exploit the administration of DM151 with nanoparticle-encapsulated TMZ. For example, some selenium nanoparticles (SNPs) loaded with TMZ and coated by Eudragit^®^ RS100 and chitosan were shown to slowly release TMZ within 5 h [57]. The co-administration of DM151 with such nanomaterial would mimic the pre-incubation, providing an attractive therapeutic strategy.

## 3. Materials and Methods

### 3.1. Materials

#### 3.1.1. Chemicals

Reduced GSH, 1-chloro-2,4-dinitrobenzene (CDNB), ampicillin, sodium dodecyl sulfate (SDS), and the chromatographic material Sepharose CL-6B were purchased from Sigma-Aldrich, St. Louis, MO, USA (Merck). Resveratrol, colchicine, gallic acid, safranal, coumaric acid, naringenin, polydatin, taxifolin hydrate, curcumin, quercetin, epigallocatechin gallate, and ellagic acid were purchased from Sigma-Aldrich, USA (Merck). Ethanol, methanol, and dimethyl sulfoxide (DMSO) were purchased from Scharlau (Sentmenat, Spain). The media/agents for cancer cell lines were purchased from Thermo Fisher Scientific (Waltham, MA, USA). Human malignant glioblastoma cells U-251 MG and U-87 MG (human malignant glioma cells) were from the cell bank of the Institute of Biosciences & Applications, NCSR “Demokritos”. The (3-[4,5-dimethylthiazol-2-yl]-2,5-diphenyl-tetrazolium bromide) reagent (MTT) was purchased from Applichem (Darmstadt, Germany).

#### 3.1.2. Bacterial Strains and Plasmids

*Escherichia coli* (*E. coli*) strain BL21 (DE3) was used as the expression host for the production of the recombinant enzyme hGSTA4-4. The plasmid pEXP5-CT/TOPO^®^ was supplied by Invitrogen, (Waltham, MA, USA). The sequence coding for hGSTA4-4 was obtained from Harvard Institute of Proteomics (Harvard PlasmidID: HsCD00327660). The In-Fusion HD Cloning Kit was obtained from Takara Bio USA, Inc. (San Jose, CA, USA). The pETite plasmid was purchased from Lucigen (Middleton, WI, USA).

#### 3.1.3. Curcumin Derivatives

The monocarbonyl curcumin derivatives were synthesized and reported previously by our group [37].

### 3.2. Methods

#### 3.2.1. Cloning, Expression, and Purification of hGSTA4-4 from Recombinant *E. coli* Cells

PCR used to amplify the full-length ORF of the hgsta4 gene, which was cloned using the In-Fusion HD Cloning Kit (Takara Bio USA, Inc., San Jose, CA, USA). PCR-primers [5′-GAA GGA GAT ATA CAT ATG GCA GCA AGG CCC AAG-3′ (forward primer) and 5′-GTG ATG GTG GTG ATG ATG TGG CCT AAA GAT GTT GTA GAC G-3′ (reverse primer)] were designed according to the hgsta4 gene sequence (Harvard PlasmidID: HsCD00327660). The PCR reaction was carried out in a total volume of 25 μL, containing: 12.5 μL Clone Amp HiFi PCR Premix, 10 μM forward and reverse primer, 20 ng template DNA, and 9.5 μL H_2_O. The PCR protocol was initiated by an initial denaturation step at 98 °C for 4 min. The PCR protocol comprised 35 cycles of 10 s at 95 °C, 15 s at 64 °C (annealing temperature), and 10 s at 72 °C (extension temperature). A final extension time at 72 °C for 10 min was performed after the 35th cycle. The resulting PCR amplicon was ligated into the pETite (Lucigen, Middleton, WI, USA) expression vector. The resulting expression construct pETite-hGSTA4-6His was verified by Sanger sequencing and was used to transform *E. coli* BL21 (DE3) cells. Purification of hGSTA4-4 was achieved by metal ion affinity chromatography on a Ni-IDA-Sepharose column. Cell lysis was performed using ultrasound in a 50 mM NaH_2_PO_4_ buffer (pH 8.0) containing 300 mM NaCl and 10 mM imidazole. The crude enzyme extract was centrifuged, for removing cell debris, and the clear supernatant was loaded onto a Ni^2+^-IDA-Sepharose column equilibrated with 50 mM NaH_2_PO_4_ buffer (pH 8.0) containing 300 mM NaCl and 10 mM imidazole. The column was washed with equilibration buffer (5 mL), following by Wash Buffer 1 (50 mM NaH_2_PO_4_ buffer, pH 8, containing 300 mM CH_3_COONa), Wash Buffer 2 (50 mM NaH_2_PO_4_ buffer, pH 8, containing 300 mM CH_3_COONa and 20% *w*/*v* glycerol), and Wash Buffer 3 (50 mM NaH_2_PO_4_ pH = 6.3, containing 300 mM CH_3_COONa). The enzyme was eluted in two fractions (1 mL each) in 50 mM NaH_2_PO_4_ buffer pH 8.0, containing 300 mM NaCl and 150 mM, and in two fractions (1 mL each) containing 250 mM imidazole. Protein concentration was determined according to the Bradford assay using bovine serum albumin as a standard.

#### 3.2.2. Enzyme Assays and Inhibition Studies

hGSTA4-4 activity was determined by monitoring (at 25 °C) the formation of the conjugate between CDNB and GSH at 340 nm (ε = 9600 L·mol^−1^·cm^−1^) for 120 s, as previously described [58]. One unit of enzyme activity is defined as the amount of enzyme that produces 1.0 μmol of product per minute under the assay conditions. The tested natural products and monocarbonyl curcumin derivatives were dissolved in DMSO (100 μΜ) and added to the assay mixture (the volume of DMSO was maintained at a 2% *v*/*v* final concentration). Initial velocities were determined in triplicates and were corrected for spontaneous reaction rates, when necessary. For the determination of IC_50_ values for the most potent inhibitors, the assay mixture was identical to that described above, in the presence of different concentrations of inhibitors. The IC_50_ values were determined from a graph of the remaining enzyme activity (%) against inhibitor concentration. The computer program GraphPad Prism version 8 (GraphPad Prism Software, Inc., Boston, MA, USA) was used for producing kinetic graphs and determining apparent kinetic parameters/constants and IC_50_ values.

#### 3.2.3. Kinetic Inhibition Analysis

Initial velocities (25 °C) for the hGSTA4-4-catalyzed reaction with CDNB as a variable substrate were determined in reaction mixtures (1 mL total volume) containing potassium phosphate buffer (100 mM, pH 6.5), 2.5 mM GSH, and different concentrations of CDNB (typically 14–1000 μM) in the absence and presence of inhibitors (0–50 μM). Initial velocities for the hGSTA4-4-catalyzed reaction with GSH as a variable substrate were determined in reaction mixtures (1 mL total volume) (25 °C) containing potassium phosphate buffer (100 mM, pH 6.5), 1 mM CDNB, and different concentrations of GSH (typically 37.5–3750 μΜ) in the absence and presence of inhibitors ellagic acid and DM151 (0–50 μM).

#### 3.2.4. Molecular Docking

The binding poses of the four most potent inhibitors ellagic acid, DM151, DM101, and DM100 were generated with the molecular docking protocol based on the CANDOCK algorithm [47] and the radial-mean-reduced scoring function at a cutoff radius of 6 Å from each atom of the ligand (RMR6). The X-ray crystal structure of hGSTA4-4 from the Protein Data Bank (PDB ID: 3IK7, chain A) was chosen for the molecular docking analysis. Avogadro [59] was used to prepare 3D structures of the four studied inhibitors, which were geometrically optimized in the Gaussian 16 [60] program using the Hartree–Fock method and 6-31G(d) basis set. The binding poses of the studied inhibitors were selected after visual inspection in PyMOL [61] based on the lowest docking score values. The detailed interactions of the inhibitors, ellagic acid, DM151, DM101, and DM100, with hGSTA4-4 were evaluated using the Protein Ligand Interaction Profiler (PLIP) [48].

#### 3.2.5. *In Vitro* Cytotoxicity with MTT Assay

U-251 and U-87 MG cells were grown in Dulbecco’s modified Eagle’s growth medium (pH 7.4) supplemented with 10% FBS, 100 U/mL penicillin, 2 mM glutamine, and 100 μg/mL streptomycin. Cell cultures were maintained in flasks and grown at 37 °C in a humidified atmosphere of 5% CO_2_. Subconfluent cells were detached using 0.05% (*w*/*v*) trypsin−0.25% (*w*/*v*) ethylenediaminetetraacetic acid solution at a subcultivation ratio of 1:2 to 1:5. The *in vitro* cytotoxicity of DM148 and DM151 was determined using the MTT colorimetric assay. Cells were seeded in 96-well plates (8 × 10^3^ cells/well in 100 μL culture medium) and grown overnight at 37 °C in a 5% CO_2_ incubator. Exponentially growing cells were incubated for 48 h with various concentrations ranging between 10^−3^ and 10^−8^ M of each compound. The final DMSO concentration never exceeded 0.2%. The medium was then removed and replaced with 100 μL MTT solution (1 mg/mL). After 4 h incubation, the solution was aspirated, formazan crystals were solubilized in 100 μL DMSO, and the absorbance was recorded at 540 nm (Tecan well plate reader). The same procedure was followed for the TMZ sensitization experiments. DM148 and DM151 were tested at a final concentration of 10 μΜ, whereas TMZ was tested at a final concentration of 150 μM. Data were analyzed using the GraphPad Prism 5.0 software.

## 4. Conclusions

The present work revealed that ellagic acid was the strongest inhibitor among all natural products and the monocarbonyl curcuminoids tested. Molecular docking studies confirmed this finding, showing that ellagic acid exhibited the lowest docking score among all studied hGSTA4-4 inhibitors. Four *π-π* stacking interactions with Phe220 and Phe111, two hydrogen bonds with Tyr-9, and a *π*-cation interaction with Arg15 are predicted to be the main structural determinants for the strong inhibitory effect of ellagic acid. DM151, a cyclohexanone monocarbonyl demethylated curcumin derivative, was the second-best hGSTA4-4 inhibitor tested, exhibiting the second-lowest docking score value. Furthermore, DM151 exhibited the highest cell-killing effect against two commonly used GBM cell lines, whereas the least active monocarbonyl hGSTA4-4 inhibitor was far less cytotoxic. This may imply that hGSTA4-4 inhibition potency may contribute to the glioblastoma cell cytotoxicity action. The potential role of hGSTA4-4 in GBM chemotherapy was further supported by the effective TMZ sensitization of cells treated with the potent monocarbonyl inhibitor. Further studies are necessary to fully characterize the potential of hGSTA4-4 as a therapeutic target for glioblastoma monotherapy or synergistic therapy, and to establish the cellular and molecular consequences of its inhibition to maximize the anti-glioblastoma therapeutic outcome.

## Figures and Tables

**Table 3:** IC_50_ values and inhibition constants (K_i_) obtained by kinetics inhibition studies of the most potent inhibitors, ellagic acid and DM151, towards hGSTA4-4.

Inhibitor	IC_50_ (μΜ)	Variable Substrate	Type of Inhibition	Inhibition Constant K_i_ (μΜ)	Inhibition Constant K_i_′ (μΜ)
Ellagic acid	0.44 ± 0.01	CDNB	competitive	0.63 ± 0.03	-
		GSH	competitive	0.39 ± 0.02	-
DM151	2.4 ± 0.1	CDNB	mixed	4.1 ± 0.5	0.53 ± 0.03
		GSH	competitive	0.98 ± 0.11	-

**Table 4:** The lowest docking score values of the four studied inhibitors in complex with hGSTA4-4.

Ιnhibitors	Docking Score Values (Arbitrary Units)
Ellagic acid	−45.18
DM151	−44.59
DM101	−36.37
DM100	−33.37

**Table 5:** Calculated IC_50_ (μΜ) values (± SD) for U-251 MG and U-87 MG cells cultured with DM148 and DM151 for 48 h. All data are representative of at least three independent experiments.

	IC_50_ (μM)	
	U251-MG	U87-MG
DM148	89.21 ± 3.41	91.09 ± 5.64
DM151	25.57 ± 4.83	18.95 ± 5.81

## Data Availability

All relevant data are included in the article.
